# Physicians and the Clergy

**Published:** 1848-09

**Authors:** 


*Physicians and the Clergy.*—At the annual convention of the Connecticut Medical Society, May, 1848, the following preamble and resolution were submitted by one of the members, and laid upon the table for consideration at the next convention:—

"Whereas, it is believed to be the custom of the regular physicians of the State, at the present time, to render *medical services* to clergymen and their families gratuitously;

"And whereas it is believed that as a class of citizens, (their education, intelligence, and moral standing considered,) they do more than any other class in the community to embarrass the legitimate influence of the medical profession; Therefore,

"*Resolved*—That as a rule we adopt the practice of charging clergymen the same fees as other citizens, except in cases of misfortune or inability, which would render it burdensome to make a just compensation for services rendered."

The subjects involved in the foregoing preamble and resolution have, of late, occasioned considerable discussion in medical journals. We have made the citation as a text for a few remarks.

We cannot accord in the propriety of the resolution as a consequence of the facts stated in the second paragraph of the preamble. Admitting that, as a class of citizens, clergymen have done much to impair public confidence in the medical profession, it does not strike us that, *on that account*, we should adopt a new line of conduct toward them, as respects charging fees, or other measures. This would be acting with a spirit of retaliation, and would compromise the self-respect of the profession. Besides, the offence charged upon the clergy is not justly attributable to them as a body, but applies only to a certain portion, for whose actions it would be as unjust to hold the whole class responsible, as it is for the public to hold our profession responsible for the ignorance and misconduct of individual practitioners of medicine. If there be any good reasons why the clergy should not be charged for medical services, it should by no means be insisted that they are thereby placed under such obligations to the profession, that they are bound, for this reason, to say or do nothing to prejudice its character and interests. It would be indelicate, on our part, to urge any such claim, and involves a concession of weakness which, for one, we are unwilling to acknowledge. We should be slow to concede that the medical profession is in danger of suffering serious injury, or inconvenience, from the efforts of those clergymen who throw the weight of their influence in the scale of empiricism. The public have more reason to find fault on this score than the profession. We would not, in short, circumscribe, in the least, the freedom of the clergy as respects our profession. It is their prerogative, as it is the prerogative of every one, to think and speak of us they please; they have an indisputable right to indulge in medical delusions, and to recommend quack nostrums. These are among the inalienable privileges of freemen—privileges for the exercise of which, certainly, the clerical profession involves no disqualification!

The question, then, turns, not on what is due from the clergy to us by way of reccompense for services professedly gratuitous, but solely on its abstract merits. Let us consider, briefly, the reasons *pro.* and *con.* which bear upon it. We will say here, that our opinion is averse to the exemption of the clergy from medical fees, as a general rule. We are in favor of adopting the custom of making charges against them, as against others. This course, in the first place, is but fair toward them. It places them in an independent position with respect to us. They ought not, as we have just remarked, feel trammeled by obligation to our profession, which shall either compromise their opinions respecting us, or their perfect freedom of speech and actions in regard to us. Such obligations, it should be considered, may not only prove a restraint upon their sayings and doing against us, but tend to impair the force of their efforts in our behalf. The countenance of the clergy would be less open to suspicion of being unbiassed, if they stood in an independent ralation to us, and would, therefore, be more valuable in the estimation of the captious.

In the second place, as our friend Prof. Coventry has remarked in a communication on the subject of medical reform, publiehed some years since in the New-York Journal of Medicine, the Clergy would place a higher value upen medical services if, like others, they were accustomed to regard them by the light of a respectable pecuniary equivalent. We are inclined, with Prof. C., to think that the want of confidence in legitimate medicine which prevails among the clergy, is, in no small degree, attributable to the fact, that they are accustomed to receive medical services gratuitously. Cash valuation is the general standard by which most benefits are appreciated, and, it is highly probable, this consideration has its effect with clergymen, as with others.

A third reason, and one which appears to us all-sufficient of itself, is, that, as a general rule, clergymen are better able to pay for medical services, than physicians to bestow them gratuitously. The salary of a clergyman, it is true, seldom, if ever, enables him to accumulate wealth, and wealth is occasionally acquired by the practice of medicine; but, if we do not err, the average pecuniary cempensation of the clergy is above the average compensation of physicians. The former, too, have this advantage:—they generally enjoy a respectable income directly upon entering their profession, while the physician, for several years, is generally compelled to struggle with pecuniary embarrassments. It not infrequently happens that the physician gives his services to clergymen in easy circumstances, while he is himself harassed with the anxieties of providing for his family. Appreciating the injustice of this, clergymen often insist upon returning a pecuniary equivalent. Such instances have fallen under our own knowledge and experience. Looking at the abstract merits of the matter, it certainly seems to be a fair conclusion, that if clergymen are better able to pay for medical services, than physicians are to forego compensation, it is due to the latter, and but justice to both parties, that such services should not be gratuitously rendered.

Let us look, now, at some of the reasons which may be adduced to sustain an opposite opinion. It may be said that the clergy have usually moderate incomes. We have just disposed of that objection. The medical profession, generally, are as poorly paid as the clerical. It may be added, that it is not the fault of physicans that the clergy are not better paid than they are. In determining the salary of a clergyman as adequate to his wants, all his expenses should be considered, and his parishoners should include, and make proper allowance for medical, as well as other bills. No other class of the community than the medical, act upon the principle that the clergy are not to be charged. The grocer, butcher, tailor, dry-goods seller, etc., do not expect to abate in the price of their commodities in consequence of the clerical profession of the purchaser; why should this be required of the physician? There are, of course instances in which it is not less a duty, than a privilege, to render services to clergymen, as to others, without charge. Their circumstances may call for this exemption, or it may be done as a tribute of personal consideration or friendship. We are not contending that in their professional relations to individual members of the clerical calling, physicians may not be as liberal and friendly as they please, but we are considering the propriety of a principle, or general rule.

Another reason which may be assigned is, that proper respect for the clergy demands that medical services should be gratuitously bestowed.

But we may ask, why is this particular method of testifying respect applicable solely to medical men? It is not considered disrespectful for every other class not to vary in their dealings with clergymen from their general principles of doing business, why should a similar course be disrespectful in a physician? If one will reflect a moment upon these inquiries, the force of this reason will, as we think, be found to consist wholly in its speciousness.

No other reason occurs to us, except that it may be considered good policy to conciliate, in this way, the good opinion of tbe clergy, in order to secure their influence. As applicable to the profession at large, this reason has not even the merit of speciousness. If medicine, and the medical profession, cannot be sustained without dependence on extrinsic support, let them go. We ask nothing beyond a fair appreciation of their intrinsic qualities. As applicable to individuals, the reason is even more objectionable. A course which institutes claims for professional patronage, other than those springing from professional and moral worth, is surely to be scouted by every true lover of his profession, and nothing can be more contemptible than an attempt to secure the favor of a religious congregation by clinging to the skirts of its pastor. We should feel that we were insulting our readers were we to dwell longer upon this topic.

We have been led to indulge in this brief discussion, by having noticed several articles in our exchanges, which have suggested reflections upon the subject. The truth is, the medical profession sustains a disproportionate share of public labor, for which no compensation is received, and but little credit. We are expected to be ever prompt and ready to devote our services to the poor, and public sentiment is shocked at any want of alacrity, or at any intimation that the *onus* should be more equally distributed. Gratuitous services to the clergy are virtually rendered to the public, inasmuch as the public are bound to afford an adequate, independent support to those who minister in holy things. Medical men, as a class, probably contribute their full quota for the support of the ministry, and other public objects, exclusive of services of a professional character. Our profession, as all must admit, is one of constant labor and anxiety. We do not, like the clergy, have frequent opportunities for relaxation. The pastor may annually leave his duties for a season, for health and enjoyment. It is seldom that the medical practitioner can in this way recruit his strength and energies. His vocation requires him to be ever at his post, or, if he takes a brief period of relaxation, it requires a sacrifice he can often illy afford to make. It is one of the highest recommendations of our calling, that it affords so many opportunities for charitable actions. We would not relinquish an iota of its character as a humane profession. But we think the public has yet much to learn respecting the reciprocal duties which exist between it and the profession. Were these duties better understood, the pnblic would be, if not more considerate, at least more gracious in some of its requisitions.

